# Molecular and morphological evidence reveals four new species of the genus *Lepiota* (*Agaricales*, *Verrucosporaceae*) in Hebei Province, China

**DOI:** 10.3897/mycokeys.142.200915

**Published:** 2026-10-26

**Authors:** Qi An, Xi-Ya Zhao, Rui-Min Li, Wan-Rui Shui, Wen-Bo Zhang, Yi-Man Liu, Yu-Jiao Zhang, Li-An Wang, Xuan Zhao, Mei-Ling Han

**Affiliations:** 1 Guangxi Key Laboratory of Health Care Food Science and Technology/ Guangxi University Engineering Research Center for Taro Preservation and High-value Utilization, Hezhou University, Hezhou 542899, China Guangxi Key Laboratory of Health Care Food Science and Technology/ Guangxi University Engineering Research Center for Taro Preservation and High-value Utilization, Hezhou University Hezhou China; 2 School of Food and Biological Engineering, Hezhou University, Hezhou 542899, China School of Food and Biological Engineering, Hezhou University Hezhou China; 3 College of Life Science, Langfang Normal University, Langfang 065000, China College of Life Science, Langfang Normal University Langfang China; 4 College of Life Sciences, Hebei Normal University, Shijiazhuang 050000, China College of Life Sciences, Hebei Normal University Shijiazhuang China; 5 Department of Ecology and Environment of Hebei Province, Shijiazhuang 050051, China Department of Ecology and Environment of Hebei Province Shijiazhuang China

**Keywords:** Lepiotaceous fungi, *
Lepiota
*, morphology, multigene, phylogeny

## Abstract

Four new species of *Lepiota* collected from Hebei Province, northern China, namely *Lepiota huangyangshanensis*, *L. maojingbaensis*, *L. varia*, and *L. xiaowutaishanensis*, are described based on morphological characteristics and molecular data. *Lepiota huangyangshanensis* is characterized by a cream to straw-yellow pileus with olivaceous buff to straw-yellow squamules, small ellipsoid to amygdaliform basidiospores, narrowly clavate to clavate cheilocystidia, and pileus and stipe coverings composed of chains of either spherical or cylindrical cells. *Lepiota maojingbaensis* features a cream to pale clay-buff pileus, a clay-pink to clay-buff stipe covered with white to cream tomentum in the lower half, a trichodermal pileus covering, and truncate to spurred basidiospores. *Lepiota varia* exhibits a cream to flesh-pink pileus with squamules ranging from flesh-pink to brownish vinaceous to rose, a cream to clay-buff stipe with buff, flesh-pink to fawn squamules, a trichodermal pileus covering, and truncate to spurred basidiospores. *Lepiota xiaowutaishanensis* has a white, flesh-pink to clay-pink pileus, a flesh-pink to fawn stipe, a hymeniform pileus covering, and ellipsoid, broadly ellipsoid to oblong basidiospores. Phylogenetic analyses based on a four-locus dataset (ITS, nrLSU, *RPB2*, mtSSU) assigned the four new species to three well-supported sections: sect. *Eriophorae*, sect. *Stenosporae* and sect. *Cristatae*. Furthermore, subsect. *Paralilaceae* was raised to the rank of section, as it did not form a monophyletic clade within sect. *Lilaceae* phylogenetically.

## Introduction

The genus *Lepiota* (Pers.) Gray is a species-rich genus of litter-decomposing fungi in *Agaricales* (Kalichman et al. 2020; Asif et al. 2022; Ahamed et al. 2023, 2024). It is widely distributed across tropical to temperate regions worldwide and mainly grows in forest and grassland soils rich in humus (Sarawi et al. 2025a). Traditional infrageneric classification mainly relies on the microstructure of the pileus covering and basidiospore characteristics (Knudsen 1980). Under the modern classification concept, the agaricoid species belonging to the genus *Lepiota* are characterized by basidiomata with universal or partial veils, free lamellae, whitish spore prints, predominantly dextrinoid basidiospores, and clamp connections (Vellinga 2003).

Historically, *Lepiota* has been accommodated within *Agaricaceae* Chevall. (Pegler 1986; Vellinga 2001a; Yang et al. 2019); however, recent taxonomic studies recognize this lineage as a distinct family, *Verrucosporaceae* Jülich (Kalichman et al. 2020; Salichanh et al. 2024; Yang et al. 2024; Qu et al. 2025). Substantial variation in macromorphology, coloration, and microscopic characters makes the taxonomic delimitation of *Lepiota* species particularly challenging (Vellinga 2003, 2004; Sysouphanthong et al. 2011, 2020; Li et al. 2025a; Sarawi et al. 2025b). Historically, there has been considerable taxonomic disagreement regarding the infrageneric classification of *Lepiota* and the taxonomic status of *Echinoderma* (Kühner 1936; Singer 1975; Bon 1981; Candusso and Lanzoni 1990; Bon 1993; Vellinga 2001a, 2003, 2004; Hausknecht and Pidlich-Aigener 2005; Zecchin and Bizzi 2008). However, recent multilocus phylogenetic analyses (ITS + nrLSU + *RPB2* + mtSSU) by Sarawi et al. (2025a) provided significant clarity, establishing *Echinoderma* as an independent genus and resolving *Lepiota* into seven stable sections: sect. *Eriophorae* (Bon) Reschke & Sarawi, sect. *Lepiota* (Pers.) Gray, sect. *Fuscovinaceae* Bon & Candusso, sect. *Stenosporae* J.E. Lange ex Reschke & Sarawi, sect. *Cristatae* Kühner ex Wasser, sect. *Helveolae* (Bon & Boiffard) Bon, and sect. *Lilaceae* Bon.

As of 22 September 2026, approximately 610 species of the genus *Lepiota* are recognized according to the Index Fungorum and MycoBank databases. This figure represents a current database estimate and may include taxa that have not yet been formally transferred to other genera. Approximately 85 species have been reported from China, including 25 new species originally described from the country (Naseer et al. 2022; Liang et al. 2023; Li et al. 2025b; Li et al. 2026; Zhou and Bau 2026). Of these 25 taxa, eight have been recorded in northern China (one in Shanxi, six in Jilin, and one in both Jilin and Beijing) (Mao et al. 2023; Zhou and Bau 2026). These species are classified under sect. *Stenosporae* (seven species) and sect. *Cristatae* (one species), suggesting that the diversity of *Lepiota* in northern China remains significantly underexplored.

Consequently, the current study, conducted as part of a comprehensive survey of macrofungal diversity in Hebei Province, northern China, specifically aimed to investigate the *Lepiota* flora of this area in detail. Through detailed morphological comparisons and multilocus phylogenetic analyses, four new species belonging to the *Lepiota* sect. *Stenosporae*, sect. *Cristatae*, and sect. *Eriophorae* were uncovered. These new taxa are formally described and illustrated herein, thereby enhancing our understanding of the fungal diversity in this understudied region.

## Materials and methods

### Sample collection and morphological studies

Fresh basidiomata were collected from natural habitats in Hebei Province, China, and deposited at the herbaria of Hezhou University (HZC) and Langfang Normal University (LFNC). Macromorphological descriptions, such as color, size, and stipe characteristics, were based on on-site records of fresh specimens. Color descriptions in this study strictly followed the fungal color standards established by Petersen (1996), ensuring consistency and comparability in morphological characterizations. Specimen sections were mounted in 10% potassium hydroxide solution (KOH) and Congo red, while cotton blue (CB), Melzer’s reagent (IKI), and brilliant cresyl blue were also used as auxiliary reagents for the identification of specific structures. The notation “[*n*/*m*/*p*]” is defined as ‘*n*’ basidiospores randomly selected from ‘*m*’ basidiomata of ‘*p*’ collections. Twenty basidia and cystidia were measured from each collection. Basidiospore dimensions are expressed in the format (*a*–)*b*–*c*(*d*) × (*a*–)*b*–*c*(*d*) for length × width, where (*a*) denotes the minimum value, the range *b*–*c* denotes 90% of the calculated values, and *d* denotes the maximum value; *X_mm_* denotes the mean of basidiospore means (± SD); *Q* represents the length/width ratio of basidiospores; *Qav* denotes the average *Q* of all tested basidiospores; and *Qm* is *Qav* ± SD (Rehman et al. 2024; Tarafder et al. 2026).

### DNA extraction, polymerase chain reaction (PCR) amplification, and sequencing

Genomic DNA was extracted from the dried specimens using the CTAB rapid plant genome extraction kit-DN14 (Aidlab Biotechnologies Co., Ltd., Beijing) according to the manufacturer’s instructions, with appropriate protocol optimizations described by Han et al. (2016, 2026).

Polymerase chain reaction (PCR) was performed to amplify sequences of the ITS, nrLSU, mtSSU, and *RPB2* regions. Primers ITS5 and ITS4 were used for the internal transcribed spacer (ITS) (White et al. 1990), while LR0R and LR7 were used for the nuclear ribosomal large subunit (nrLSU) (Vilgalys and Hester 1990). The mtSSU gene was sequenced using primers MS1/MS2, and the *RPB2* gene was targeted using RPB2-6F/RPB2-7R for QA-33 and RPB2-6F/RPB2-7cR for other specimens (White et al. 1990; Matheny 2005, 2006; Han et al. 2016). ITS, nrLSU, mtSSU, and *RPB2* were amplified in 50 µL reactions containing 25 µL of 2× Taq PCR Master Mix (Aidlab Biotechnologies Co., Ltd., Beijing, China), 20 µL of ddH_2_O, 1.5 µL each of forward and reverse primers (10 µM), and 2 µL of DNA. The PCR procedure followed that described by Han et al. (2016, 2026). The PCR products were purified and sent to the Beijing Genomics Institute (China) for direct sequencing. All newly generated sequences were submitted to the GenBank database (https://www.ncbi.nlm.nih.gov/genbank), and information on all sequences used in the phylogenetic analyses is listed in Table 1.

**Table 1.** List of species, specimens, and GenBank accession numbers of sequences used in this study.

**Species**	**Voucher**	**Origin**	**GenBank accession numbers**				**References**
			**ITS**	**LSU**	***RPB2***	**mtSSU**	
*Cystolepiota bucknallii*	SeSa12	Germany	OL527684	PP594631	–	–	Sarawi et al. 2022, 2025b
*Lepiota alba*	KUN-HKAS 90371	China	MN810115	MN810075	MN820946	–	Hou and Ge 2020
*L. albofloccosa*	MAA01	India	OP954870	OP954873	–	–	Pious and Kumar 2025
*L. albogranulosa*	SeSa342	Benin	PP594605	PP594728	PP841237	PQ152809	Sarawi et al. 2025a, 2025b
	T14/LAH:10152012, Holotype	Pakistan	LK932284	–	–	–	Qasim et al. 2015
*L. alopochroa*	MFLU 09-0178	Thailand	HQ647294	–	–	–	Sysouphanthong et al. 2011
*L. andegavensis*	8-X-1994, P.D.H. Roux 2121 (herb. Roux)	France	AY176461	–	–	–	Vellinga 2003
*L. apatelia*	BH20210810	Germany	PP594619	PP594746	PP841157	PQ152823	Sarawi et al. 2025a, 2025b
*L. asperula*	iNaturalist # 8607856	USA	MN906140	–	–	–	–
*L. atrobrunneodisca*	HSA 115, Holotype	China	OP724226	OP724232	–	OP724234	Mao et al. 2023
*L. attenuata*	HKAS50110, Holotype	China	EU681776	GU199354	MK705816	EU681826	Liang et al. 2011
*L. aureofulvella*	HNL502948	Laos	KX711967	–	–	–	Sysouphanthong et al. 2011
*L. badiopurpurea*	CP43772	Italy	PQ152692	–	–	–	Sarawi et al. 2025a
*L. bahawalnagarensis*	LAH:37787, Holotype	Pakistan	OQ658766	–	–	–	Asif et al. 2024
*L. baiyunensis*	B22052705, Holotype	China	OQ547186	OQ547188	–	OQ547190	Liang et al. 2023
*L. bengalensis*	Iqbal 825 (GDGM 45684), Holotype	Bangladesh	KU563148	KU563150	–	–	Hosen et al. 2016
*L. beninensis*	FC-21-201	Benin	PP594630	PP594755	PP841161	PQ152830	Sarawi et al. 2025b
*L. boudieri*	SeSa13	Germany	OL527685	PP594636	PP841186	PQ152758	Sarawi et al. 2022, 2025a, 2025b
	SeSa46	Germany	OL527692	PP594644	PP841252	PQ152762	Sarawi et al. 2022, 2025a, 2025b
*L. brunneoaurantia*	UEH-F0006, Holotype	Pakistan	OR464184	–	–	–	Azeem et al. 2024
*L. brunneodisca*	AKD 36/2019 (GUBH 20381), Holotype	India	ON935795	–	–	–	Roy et al. 2023
*L. brunneophora*	RITF541 (PT)	China	MK651649	–	–	–	Li et al. 2025b
	RITF548 (HT)	China	MK651650	–	–	–	Li et al. 2025b
*L. brunneopileata*	LAH:37842, Holotype	Pakistan	OQ970543	–	–	–	Rehman et al. 2024
*L. brunneosquamulosa*	HKAS:50142, Holotype	China	NR169900	–	–	KP177203	Liang et al. 2018
*L. carinii*	EMB13282005, Epitype	Italy	PQ152699	PQ152727	–	PQ152749	Sarawi et al. 2025a
*L. castanea*	RITF2403	China	MK685380	MK651688	MK705840	MK651739	–
*L. castaneidisca*	E.C. Vellinga 2300 (UC)	USA	AF391057	–	–	–	Vellinga 2001c
L. cf. eriophora	MycoMap #1183	USA	OM522679	–	–	–	–
L. cf. erythrosticta	RC MART 15.175	Martinique, France	PP594629	PP594754	PP841169	PQ152829	Sarawi et al. 2025b
*L. chiangraiensis*	MFLU 20-0197, Holotype	Thailand	MT020094	MT020099	–	–	Hyde et al. 2020
*L. cholistanensis*	LAH:35831, Holotype	Pakistan	NR173802	NG088084	–	–	Bashir et al. 2020
*L. cingulum*	20-IX-1995, M. Enderle (L)	Germany	AY176359	AY176360	–	–	Vellinga 2003
*L. citrophylla*	MFLU 09-0172	Thailand	HQ647295	–	–	–	Sysouphanthong et al. 2011
*L. clypeolaria*	SeSa95	Germany	PP594549	PP594667	–	PQ152779	Sarawi et al. 2025b
*L. coloratipes*	9-X-1991, H.A. Huijser (herb. Huijser)	Netherlands	AF391066	–	–	–	Vellinga 2010
*L. condylospora*	MFLU 090048, Holotype	Thailand	JN224822	–	–	–	Sysouphanthong et al. 2020
*L. condylospora*	HKAS49258	China	EU081937	–	MK705832	EU081992	Liang et al. 2009
*L. cortinarius*	HKAS:46095	China	EU416306	EU416307	–	–	Liang et al. 2010
*L. cremea*	OKA-TR11019, Holotype	Turkey	OL630458	–	–	–	Kaygusuz 2022
*L. cristatoides*	5-IX-1996, H.A. Huijser s.n. (herb. Huijser)	Netherlands	AY176363	AY176364	–	–	Vellinga 2003
*L. cystophoroides*	E.C. Vellinga 2142 (L)	France	AF391031	AY176365	–	–	Vellinga 2001b, 2003
*L. dolichospora*	FJAU78183H	China	PZ185053	–	–	–	Zhou and Bau 2026
	FJAU78184	China	PZ185060	–	–	–	Zhou and Bau 2026
*L. echinacea*	HZ1920915	Germany	PQ152704	PQ152731	PQ202735	PQ152740	Sarawi et al. 2025a
*L. echinella*	4-X-1998, H.A. Huijser s.n. (herb. Huijser)	Belgium	AY176366	AY176367	–	–	Vellinga 2003
*L. echinobispora*	SeSa114	Benin	PP594559	PP594677	PP841183	PQ152786	Sarawi et al. 2025b
*L. elaiophylla*	SeSa363	Germany	PP594611	PP594734	PP841243	PQ152813	Sarawi et al. 2025b
*L. elseae*	AH:40487, Holotype	Spain	NR158471	–	–	–	Caballero et al. 2015
*L. erminea*	RD21111001	Germany	PP594617	PP594744	PP841170	PQ152821	Sarawi et al. 2025b
*L. exocarpi*	PERTH:08944946	Australia	MT571655	–	–	–	–
*L. farinolens*	PR2021090701	Germany	PP594618	PP594745	PP841167	PQ152822	Sarawi et al. 2025b
*L. felina*	SeSa7	Germany	PP594523	PP594633	PP841264	PQ152736	Sarawi et al. 2025b
*L. flavonigrescens*	SeSa306	Benin	PP594597	PP594718	PP841227	PQ152806	Sarawi et al. 2025b
*L. forquignonii*	E.C. Vellinga 2284 (L)	Netherlands	AY176370	AY176371	–	–	Vellinga 2003
*L. fuscovinacea*	SeSa8	Germany	PP594524	PP594634	PP841268	PQ152738	Sarawi et al. 2025b
	S17	Germany	–	PP594739	PP841171	PQ152819	Sarawi et al. 2025b
*L. grangei*	KaiR1768	Germany	PP594624	PP594638	PP841166	PQ152826	Sarawi et al. 2025b
	FJAU78231	China	PZ185018	–	–	–	Zhou and Bau 2026
*L. griseovirens*	SeSa91	Germany	PP594548	PP594665	PP841272	PQ152777	Sarawi et al. 2025b
*L. haroonabadensis*	LAH:36801, Holotype	Pakistan	MZ172897	–	–	–	Niazi et al. 2021
*L. hongshiensis*	FJAU78254H	China	PZ185036	–	–	–	Zhou and Bau 2026
	FJAU78295	China	PZ185009	–	–	–	Zhou and Bau 2026
***L. huangyangshanensis***	**ZL-2-18, Holotype**	**China**	**PZ253851**	**PZ256940**	**PZ290195**	–	**This study**
	**Han 27, Paratype**	**China**	**PZ253852**	**PZ256941**	**PZ290196**	–	**This study**
*L. hymenoderma*	77–IX–1996, E.C. Vellinga 2017 (L)	Netherlands	AF391028	AY176376	–	–	Vellinga 2001b
*L. ignicolor*	17-X1-999, H.A. Huijser (herb. Huijser)	Netherlands	AY176472	–	–	–	Vellinga 2003
*L. ignivolvata*	SeSa101	Germany	PP594555	PP594673	PP841179	PQ152784	Sarawi et al. 2025b
*L. jacobi*	SeSa94	Germany	–	PP594666	PP841273	PQ152778	Sarawi et al. 2025b
	C-F-90220, Holotype	Denmark	PQ152711	–	–	–	Sarawi et al. 2025a
*L. jilinensis*	FJAU78248H	China	PZ185021	–	–	–	Zhou and Bau 2026
	FJAU78249	China	PZ185035	–	–	–	Zhou and Bau 2026
*L. lahorensis*	T18/LAH:10002012, Holotype	Pakistan	KT182475	–	–	–	Qasim et al. 2016
*L. lilacea*	SeSa367	Germany	PP594612	PP594735	PP841244	PQ152814	Sarawi et al. 2025b
*L. lilaceostriata*	SeSa241	Benin	PP594577	PP594697	PP841205	PQ152796	Sarawi et al. 2025b
*L.‘luteophylla’*	25-VIII-1972, H.V. Smith 284 (MICH)	USA	AY176475	–	–	–	Vellinga 2003
*L. maerimensis*	MFLU 12-2036, Holotype	Thailand	MW251839	MW251847	–	–	Tibpromma et al. 2017
*L. magnispora*	SeSa68	Austria	PP594541	PP594657	PP841263	PQ152770	Sarawi et al. 2025b
*L. mandarina*	HKAS:50028, Holotype	China	KM214811	KM214816	–	–	Liang 2016
***L. maojingbaensis***	**Han 682, Holotype**	**China**	**PZ253853**	**PZ256942**	**PZ290197**	**PZ261804**	**This study**
	**An 1, Paratype**	**China**	**PZ253854**	**PZ256943**	**PZ290198**	**PZ261805**	**This study**
*L. mengei*	UTC00253524	USA	MN810131	MN810082	MN820942	–	Hou and Ge 2020
*L. metulispora*	HMAS61859	China	EU681778	MK651673	–	–	Liang et al. 2011
*L. microstenospora*	FJAU78246H	China	PZ185011	–	–	–	Zhou and Bau 2026
	FJAU78247	China	PZ185043	–	–	–	Zhou and Bau 2026
*L. minutoflava*	SeSa283	Benin	PP594590	PP594711	PP841219	PQ152802	Sarawi et al. 2025b
*L. neophana*	RITF2402	China	MK651599	MK651661	MK705837	MK651694	–
	E.C. Vellinga 3735 (UC)	USA	GQ203809	–	–	–	Vellinga 2010
*L. ochraceofulva*	SeSa100	Germany	PP594554	PP594672	PP841178	PQ152783	Sarawi et al. 2025b
*L. ochraceosquamea*	HKAS45635 (PT)	China	MK651633	–	–	–	Li et al. 2025b
	HKAS45559 (HT)	China	MK651634	–	–	–	Li et al. 2025b
*L. omninoflava*	KUN-HKAS 106734, Type	China	MN810157	MN810092	MN820951	–	Hou and Ge 2020
*L. oreadiformis*	SeSa96	Germany	PP594550	PP594668	PP841274	PQ152780	Sarawi et al. 2025b
*L. pakistanensis*	LAH:37846, Holotype	Pakistan	OQ954776	OQ954783	–	–	Rehman et al. 2024
*L. pilodes*	SeSa379	Germany	PP594614	PP594736	PP841247	PQ152816	Sarawi et al. 2025b
*L. poliochloodes*	MFLU 081272	Thailand	HQ647296	–	–	–	Sysouphanthong et al. 2011
*L. psalion*	WU 5152, Holotype	Austria	MG581687	MG581699	–	–	Vizzini et al. 2019
	FC20103101	Germany	PP594616	PP594743	PP841160	PQ152820	Sarawi et al. 2025b
*L. pseudoasperula*	C-F-13685, Type	Denmark	PQ152710	–	–	–	Sarawi et al. 2025a
	HB60904	Germany	PQ152707	–	PQ202737	PQ152755	Sarawi et al. 2025a
*L. punaensis*	HAW:F-00256, Holotype	USA	NR173815	NG153999	OM215203	–	Stallman et al. 2020
*L. recondita*	TR gmb 01481, Paratype	Netherlands	MK508899	MK508901	–	–	Vizzini et al. 2019
*L. rhodophylla*	UC(USA-CA):1860004, Holotype	USA	NR119624	–	–	–	Schoch et al. 2014
*L. sanguineofracta*	TO-HG2916, Holotype	Italy	KF879620	–	–	–	Vizzini et al. 2019
	TO-HG2917	Italy	KF879621	–	–	–	Vizzini et al. 2014
*L. scaberula*	UC(USA-CA):1860006, Holotype	USA	NR119449	–	–	–	Schoch et al. 2014
*L. sinocastanea*	FJAU78160	China	PZ185039	–	–	–	Zhou and Bau 2026
	FJAU78171H	China	PZ185063	–	–	–	Zhou and Bau 2026
*L. sinorecondita*	HMJAU3799	China	GU199362	GU199355	–	EU681822	Liang et al. 2011
*L. sirupa*	FJAU78262	China	PZ185057	–	–	–	Zhou and Bau 2026
	FJAU78261H	China	PZ185058	–	–	–	Zhou and Bau 2026
*L. sosuensis*	JBSD:CA3, Holotype	Dominican Republic	NR184875	–	–	–	Justo et al. 2015
*L. spheniscispora*	UC(USA-CA):1860005, Holotype	USA	NR119448	–	–	–	Schoch et al. 2014
*L. spiculata*	JBSD:127426	Dominican Republic	MK696156	MK696155	MK696576	–	Angelini et al. 2020
*L. squamulodiffracta*	CA21, Holotype	Dominican Republic	KR022006	–	–	–	Justo et al. 2015
*L. subalba*	SeSa9	Germany	PP594525	PP594635	–	–	Sarawi et al. 2025b
*L. subcastanea*	HKAS:45633, Holotype	China	KM214812	KM214817	–	–	Liang 2016
*L. subgracilis*	HKAS:5802	China	EU416290	EU416291	–	–	Liang et al. 2010
*L. subincarnata*	SeSa49	Germany	PP594533	PP594646	PP841254	PQ152763	Sarawi et al. 2025b
*L. subvenenata*	170927-68, Holotype	China	MK411234	–	–	–	Zhang et al. 2019
*L. thiersii*	E.C. Vellinga 2589 (UC)	USA	GQ203817	–	–	–	Vellinga 2010
*L. thrombophora*	HKAS41003	China	EU681780	MK651687	MK705829	EU681813	–
*L. tomentella*	SeSa58	Germany	PP594536	PP594652	PP841259	PQ152768	Sarawi et al. 2025b
***L. varia***	**ZL-1-59, Holotype**	**China**	**–**	**PZ256934**	**–**	**–**	**This study**
	**ZL-2-66, Paratype**	**China**	**PZ253846**	**PZ256935**	**PZ290192**	**PZ261799**	**This study**
	**YX-2-18, Paratype**	**China**	**PZ253847**	**PZ256936**	**PZ290193**	**PZ261800**	**This study**
	**QA-33, Paratype**	**China**	**PZ253848**	**PZ256937**	**PZ290194**	**PZ261801**	**This study**
*L. vellingana*	LAH:37033, Holotype	Pakistan	HE974764	–	–	–	Nawaz et al. 2013
*L. venenata*	HKAS:101874, Holotype	China	NR160618	–	–	–	Cai et al. 2018
*L. viridigleba*	OSC:56971, Holotype	USA	NR121542	–	–	–	Schoch et al. 2014
*L. xanthophylla*	E.C. Vellinga 2240 (L)	Netherlands	AY176405	AY176406	–	–	Vellinga 2003
***L. xiaowutaishanensis***	**YX-2-7, Holotype**	**China**	**PZ253849**	**PZ256938**	–	**PZ261802**	**This study**
	**Han 5, Paratype**	**China**	**PZ253850**	**PZ256939**	–	**PZ261803**	**This study**

New sequences are shown in bold.

### Phylogenetic analyses

Sequences generated in this study were aligned with sequences downloaded from GenBank (Table 1), which were specifically selected to represent all species within the seven sections, primarily following the taxonomic framework of Sarawi et al. (2025a) and further informed by Asif et al. (2024). *Cystolepiota bucknallii* (Berk. & Broome) Singer & Clémençon was selected as the outgroup (Sarawi et al. 2022, 2025b). The alignment was performed using ClustalX (Thompson et al. 1997) and manually adjusted in BioEdit (Hall 1999).

Phylogenetic analyses of the aligned sequences were performed using maximum likelihood (ML) and Bayesian inference (BI) algorithms (Sarawi et al. 2022, 2025b; Zhang et al. 2025). ML phylogenetic trees and the best-fit partition models were inferred using RAxML-NG v. 2.0.3 (Kozlov et al. 2019), with branch support assessed using 1,000 bootstrap replicates.

ModelFinder v. 3.0.1 (Kalyaanamoorthy et al. 2017) was employed to select the best-fit nucleotide substitution model for the Bayesian analysis based on the Bayesian information criterion (BIC). BI phylogeny was inferred using MrBayes v. 3.2.7a (Ronquist et al. 2012) under the best-fit model (2 parallel runs, 4,000,000 generations) within PhyloSuite v. 2 (Zhao et al. 2025). The first one-fourth of the generations were discarded as burn-in. A majority-rule consensus tree was then computed from the remaining sampled trees. Branches with maximum likelihood bootstrap support (BS) greater than or equal to 50% and Bayesian posterior probabilities (BPP) greater than or equal to 0.95 were considered significantly supported clades, respectively. Phylogenetic trees were visualized and edited in TreeView (Page 1996) and FigTree v. 1.4.3 (https://mac.softpedia.com/get/Graphics/FigTree.shtml).

## Results

### Phylogenetic analyses

**Figure 1.**
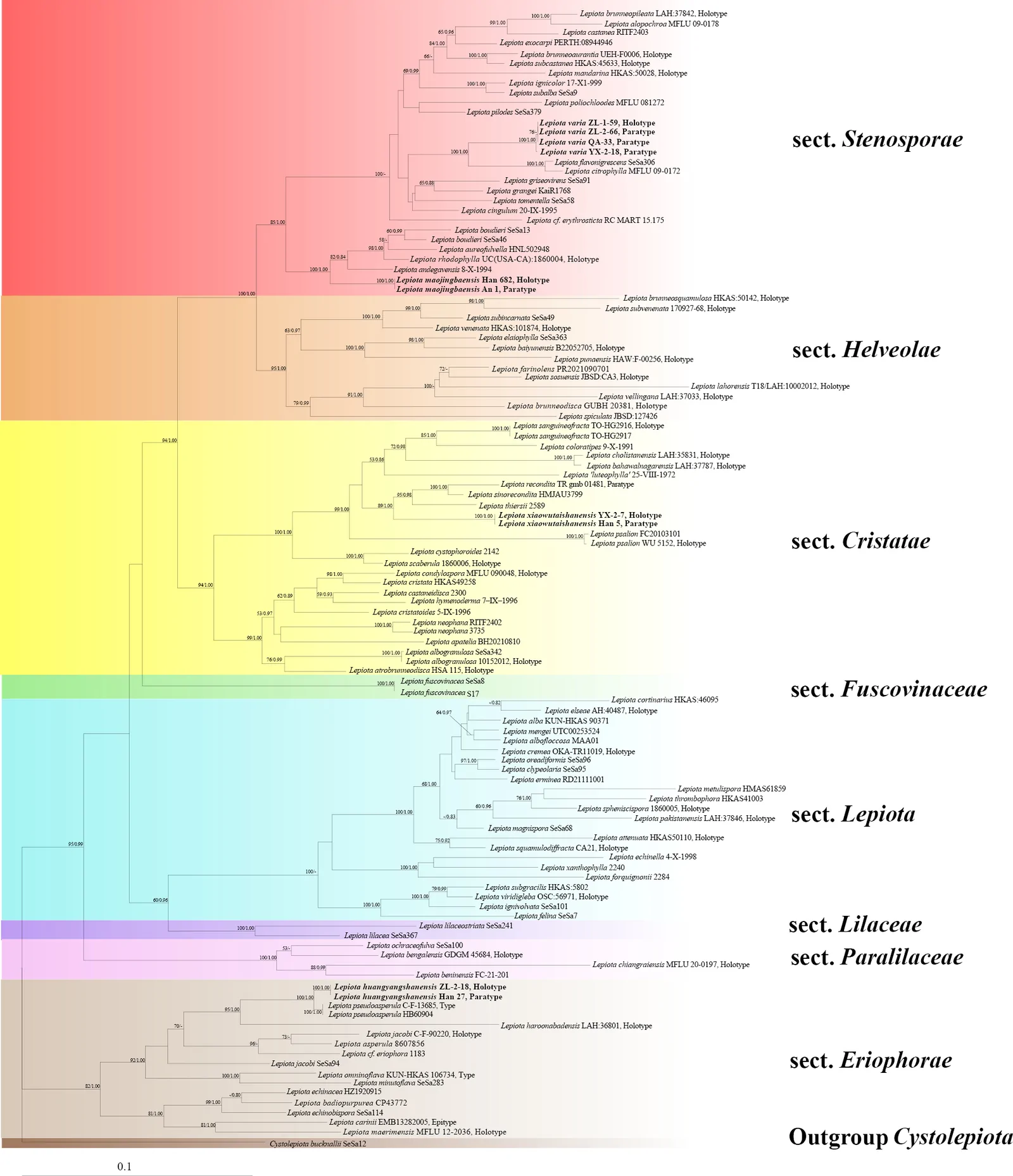
Maximum likelihood tree illustrating the phylogeny of *Lepiota* species and the outgroup based on the combined four-gene dataset (ITS + nrLSU + *RPB2* + mtSSU). Branches are labeled with maximum likelihood bootstrap support ≥ 50% and Bayesian posterior probabilities > 0.95. New species and new sequences are in bold.

The combined ITS + nrLSU + *RPB2* + mtSSU dataset included 284 sequences (113 ITS, 75 nrLSU, 47 *RPB2*, and 48 mtSSU), 33 of which were newly generated (nine ITS, 10 nrLSU, seven *RPB2*, and seven mtSSU). The best-fit partition models for the combined dataset were as follows: ITS (1–832, ML: TPM2+FU+R4+BU, BI: TPM2u+F+I+G4), nrLSU (833–2252, ML: TIM2+FU+R3+BU, BI: SYM+I+G4), *RPB2* (2253–2951, ML: TPM2+FE+IU+R3+BU, BI: TPM2u+I+G4), and mtSSU (2952–3573, ML: TPM2+FU+IU+G4m+BU, BI: TPM2u+F+I+G4). The average standard deviation of split frequencies from the BI analysis was 0.009038. The ML and BI trees are presented in Fig. 1 and Suppl. material 1, respectively.

Phylogenetic analyses (Fig. 1; Suppl. material 1) showed that the genus *Lepiota* is divided into eight well-supported clades, here recognized as sections, viz. sect. *Stenosporae*, sect. *Helveolae*, sect. *Cristatae*, sect. *Fuscovinaceae*, sect. *Lepiota*, sect. *Lilaceae*, sect. *Paralilaceae* comb. nov. (see Taxonomy section), and sect. *Eriophorae*. Phylogenetic trees inferred from BI and ML analyses yielded similar topologies, with the exception of the positions of sect. *Fuscovinaceae*, sect. *Lepiota*, and sect. *Lilaceae*. In both ML and BI trees, sect. *Lepiota* and sect. *Lilaceae* clustered together as a sister clade (BS = 60%, BPP = 0.96). In the ML tree, this clade is sister to the larger clade comprising sect. *Fuscovinaceae*, sect. *Cristatae*, sect. *Helveolae*, and sect. *Stenosporae*. In the Bayesian tree, this sister clade and sect. *Fuscovinaceae* form a larger clade (BPP = 0.83), with sect. *Fuscovinaceae* occupying the basal position. The four proposed new species, *L. varia*, *L. maojingbaensis*, *L. xiaowutaishanensis*, and *L. huangyangshanensis*, form monophyletic groups with high support (BS = 100%, BPP = 1.00). Specifically, *L. varia* and *L. maojingbaensis* are nested within sect. *Stenosporae*, while *L. xiaowutaishanensis* is recovered within sect. *Cristatae*. Additionally, *L. huangyangshanensis* is placed within sect. *Eriophorae*.

### Taxonomy

#### Lepiota huangyangshanensis

Taxon classificationFungiAgaricalesAgaricaceae

M.L. Han, L.A. Wang & Q. An sp. nov.

54844A9B-3980-565D-94BA-6BD1C8A130A6

865585

Figs 2a, 3

##### Etymology.

The specific epithet *huangyangshanensis* (Latin) refers to the location of the species in Huangyangshan National Forest Park.

##### Holotype.

China • Hebei Province, Zhangjiakou, Zhuolu County, Huangyangshan National Forest Park, 39°52'59.24"N, 115°07'24.39"E, 1,300 m asl., 6 September 2023, on ground, leg. L. A. Wang, ZL-2-18 (LFNC 0002564).

##### Diagnosis.

Similar to *L. omninoflava* Y.J. Hou & Z.W. Ge, but distinguished by its pileus covering and stipe covering composed of chains of either spherical or cylindrical cells, narrowly clavate to clavate cheilocystidia, and smaller thick-walled basidiospores (3.1–4.8 × 2.0–3.1 µm).

##### Description.

Basidiomata small, gregarious. Pileus 11–18 mm in diameter, initially campanulate to parabolic, becoming convex to applanate-convex on maturity; surface dull and dry; background cream (4A2/3) to straw-yellow (3A/B3), with denser straw-yellow (3A/B3) squamules at center becoming sparse olivaceous buff (4C4) towards margin; context white (1A1); margin decurved becoming straight at maturity. Lamellae free, white (1A1), moderately crowded, up to 1.8–2.0 mm wide, with 2–3 series of lamellulae; margin fimbriate, concolorous with faces. Stipe 23–44 × 1.5–3 mm, central, subcylindrical, hollow, with rhizomorphs; surface cream (4A2/3) to buff (4A4), with irregular cream (4A2/3) to olivaceous buff (4C4) squamules towards the base. Volva absent. Odor not distinctive.

**Figure 2.**
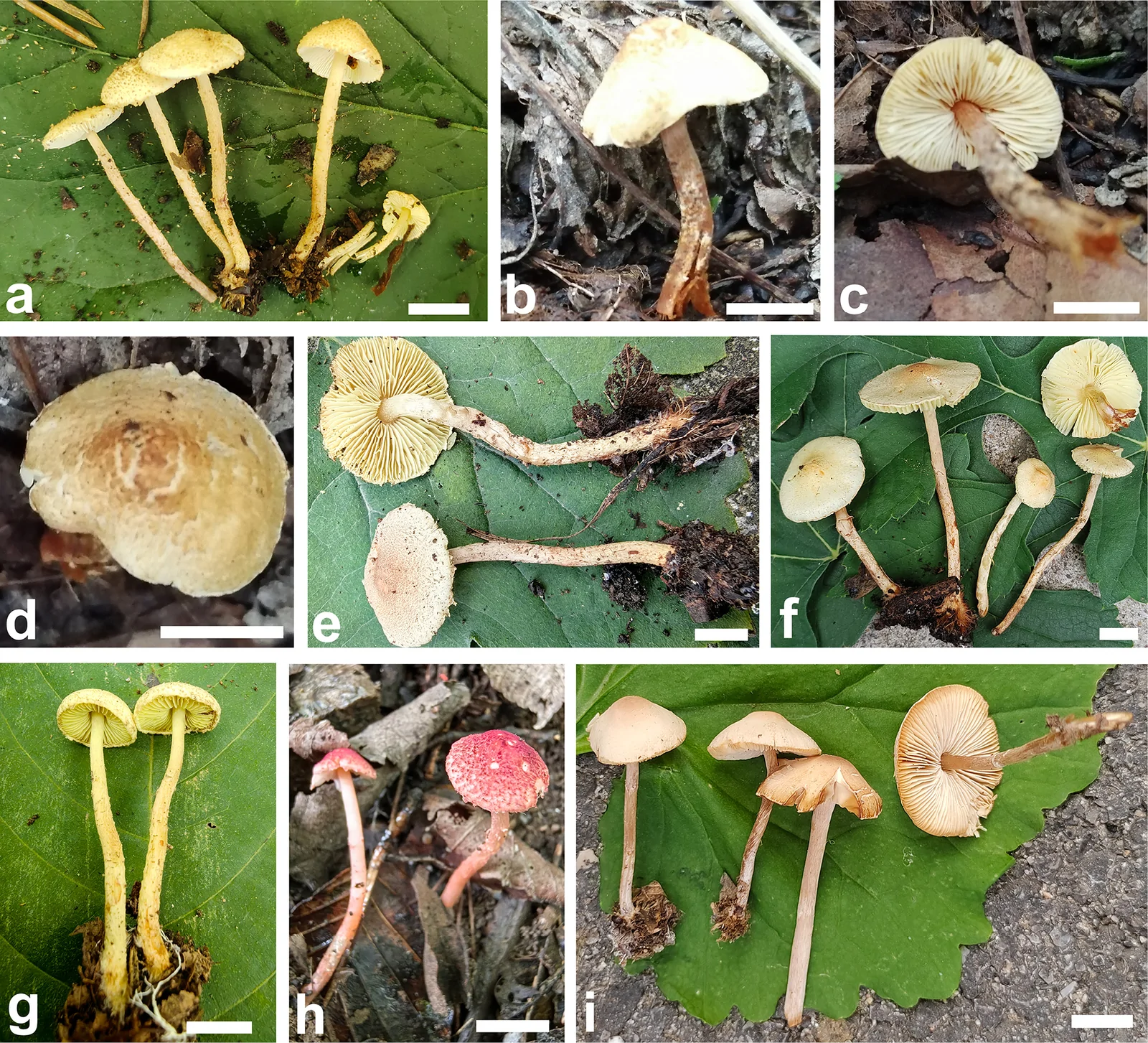
Basidiomata in the field. **a**. *Lepiota huangyangshanensis* (holotype, LFNC 0002564); **b–d**. *Lepiota maojingbaensis* (holotype, LFNC 0000682); **e**. *Lepiota varia* (holotype, LFNC 0002558); **f**. *Lepiota varia* (LFNC 0002559); **g**. *Lepiota varia* (LFNC 0002560); **h**. *Lepiota varia* (LFNC 0002561); **i**. *Lepiota xiaowutaishanensis* (holotype, LFNC 0002562). Photographs by L.A. Wang, M.L. Han, and X. Zhao. Morphology described by Q. An, X.Y. Zhao, W.R. Shui, W.B. Zhang, and M.L. Han. Scale bars: 1 cm.

Basidiospores [100/2/2] (2.8–)3.1–4.8(–5.8) × (1.5–)2–3.1(–4.1) µm, *X_mm_* = 4.02 ± 0.50 × 2.35 ± 0.42, *Q* = 1.46–2.25, *Qm* = 1.72 ± 0.15, ellipsoid in frontal view, ellipsoid or oblong in side view, hyaline, thick-walled (0.2–0.4 µm), smooth, with guttules, dextrinoid, congophilous, cyanophilic, not metachromatic, without suprahilar depression and germpore. Basidia 14–20 × 4.5–7.5 µm, clavate to broadly clavate, with guttulate contents, hyaline, thin-walled, 4-spored; sterigmata up to 4 µm long. Lamellar trama subregular; hyphae 3.5–6.8 µm wide, hyaline, thin-walled. Cheilocystidia 14.5–24 × 5.0–9.5 µm, narrowly clavate to clavate, hyaline, thin-walled. Pleurocystidia absent. Pileus trama interwoven, composed of slightly inflated, septate, 3.8–6.5 µm wide, thick-walled to solid, hyaline hyphae. Pileus covering composed of two types of agglutinated chains of terminal cells forming squamules. One type of chains formed by spherical cells, 19–41 × 9.5–35 μm, ellipsoid, subglobose to globose, often collapsed, easily detachable, thick-walled, basal cells cylindrical or narrowly clavate to clavate; the other type of chains consists of cylindrical to elongated cells, 22–52.4 × 6.7–18 μm, terminal cells with rounded or tapered apex, thick-walled. Both type of chains with pale yellowish to yellowish brown intracellular pigments. Stipe covering a cutis made up of hyphae 3.1–4.9 μm, hyaline in KOH, thin-walled to slightly thick-walled, with or without clamps, terminal elements made up of two types of agglutinated chains forming squamules. One type of terminal cells of chains formed by spherical cells, 21–55 × 9–13 μm, ellipsoid, subglobose to globose, often collapsed, easily detachable, thick-walled, basal cells cylindrical or narrowly clavate to clavate. The other type of chains consists of elongated cells, 14–38 × 11–27 μm, terminal cells with rounded or tapered apex, thick-walled. Both type of chains containing pale yellowish to brown intracellular pigments. Clamp connections present in all parts of basidiomata.

**Figure 3.**
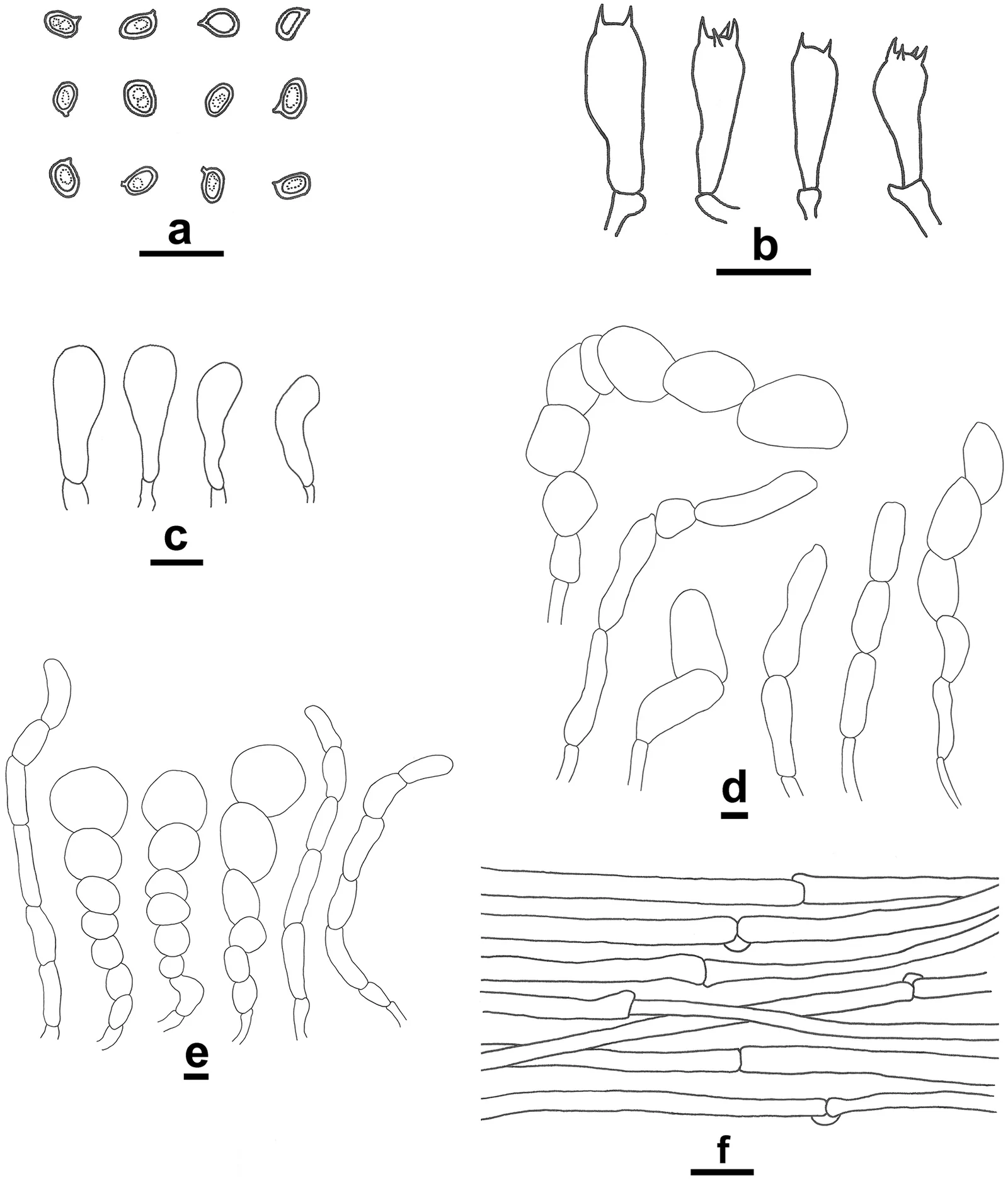
Microscopic structures of *Lepiota huangyangshanensis* (holotype, LFNC 0002564). **a**. Basidiospores; **b**. Basidia; **c**. Cheilocystidia; **d**. Terminal cells of pileus covering; **e**. Terminal elements of stipe covering; **f**. Stipe covering hyphae. Line drawings by R. M. Li and X. Y Zhao. Scale bars: 10 μm.

##### Habitat and distribution.

Solitary on the ground in mixed coniferous and broad-leaved forests dominated by *Pinus tabuliformis*, *Quercus* spp., *Betula platyphylla*, and *Populus davidiana*, Hebei province, China.

##### Additional specimens examined (paratype).

China • Hebei Province, Zhangjiakou, Zhuolu County, Huangyangshan National Forest Park, 39°52'58.6"N, 115°07'25.8"E, 1,300 m asl., 6 September 2023, on ground, leg. M. L. Han, Han 27 (LFNC 0002565).

##### Notes.

*Lepiota huangyangshanensis* and *L. pseudoasperula* (Knudsen) Knudsen formed a well-supported lineage (BS = 100%, BPP = 1.00, Fig. 1) within sect. *Eriophorae*. Morphologically, *L. pseudoasperula* resembles *L. huangyangshanensis* in possessing white lamellae and basidiospores of a similar size (4.0–5.0 × 2.2–3.0 µm). However, *L. pseudoasperula* can be distinguished by its light to pale cream pileus with dark brown scales, a white stipe bearing residual woolly tomentum with brown scales, and the absence of cheilocystidia (Malysheva 2005). *Lepiota omninoflava* has similarly colored basidiomata to *L. huangyangshanensis*; however, it is distinguished by its pileus covering composed of chains of subglobose or ellipsoidal cells, the absence of cheilocystidia, and longer (5.0–6.0 µm), thin-walled basidiospores (Hou and Ge 2020). *Lepiota keralensis* Pious & T.K.A. Kumar, a recently described species in sect. *Eriophorae* from southern India, differs from *L. huangyangshanensis* in its white pileus surface with brownish squamules, as well as its larger basidiospores (5–7 × 3–5 µm; Pious and Kumar 2025).

#### Lepiota maojingbaensis

Taxon classificationFungiAgaricalesAgaricaceae

M.L. Han, L.A. Wang & Q. An sp. nov.

FA21ADB7-5B19-55D1-BA75-38B3E10FDF70

865586

Figs 2b–d, 4

##### Etymology.

The specific epithet *maojingbaensis* (Latin) refers to the location of the species in Maojingba National Forest Park.

##### Holotype.

China • Hebei Province, Chengde, Longhua County, Maojingba National Forest Park, 41°40'39.69"N, 118°10'38.88"E, 1,450 m asl., 28 July 2019, on ground, leg. M. L. Han, Han 682 (LFNC 0000682).

##### Diagnosis.

Similar to *L. jilinensis* T. Bau & X.Y. Zhou, but differs in a cream to pale clay-buff pileus with white context and cream lamellae, smaller cheilocystidia (13–19 × 6.0–7.5 µm), and terminal cells of the pileus covering (32–65.8 × 8.2–11.5 μm).

##### Description.

Basidiomata small. Pileus 20–27 mm in diameter, conical; surface dull and dry, cream (4A2/3) to pale clay-buff (6D4), cracked at maturity; context white (1A1); margin smooth, becoming undulate with age, slightly split radially. Lamellae 3–5 mm wide, free, cream (4A2/3), crowded with 1–3 series of lamellulae. Stipe 30–45 × 4–6 mm, central, cylindrical to subcylindrical, equal or sometimes slightly broadened at base, hollow; surface clay-pink (6B/C4) and glabrous at the apical part, and clay-buff (6D4) covered with white (1A1) to cream (4A2/3) tomentum in lower half. Volva and annulus not observed. Odor farinaceous to rancid farinaceous.

**Figure 4.**
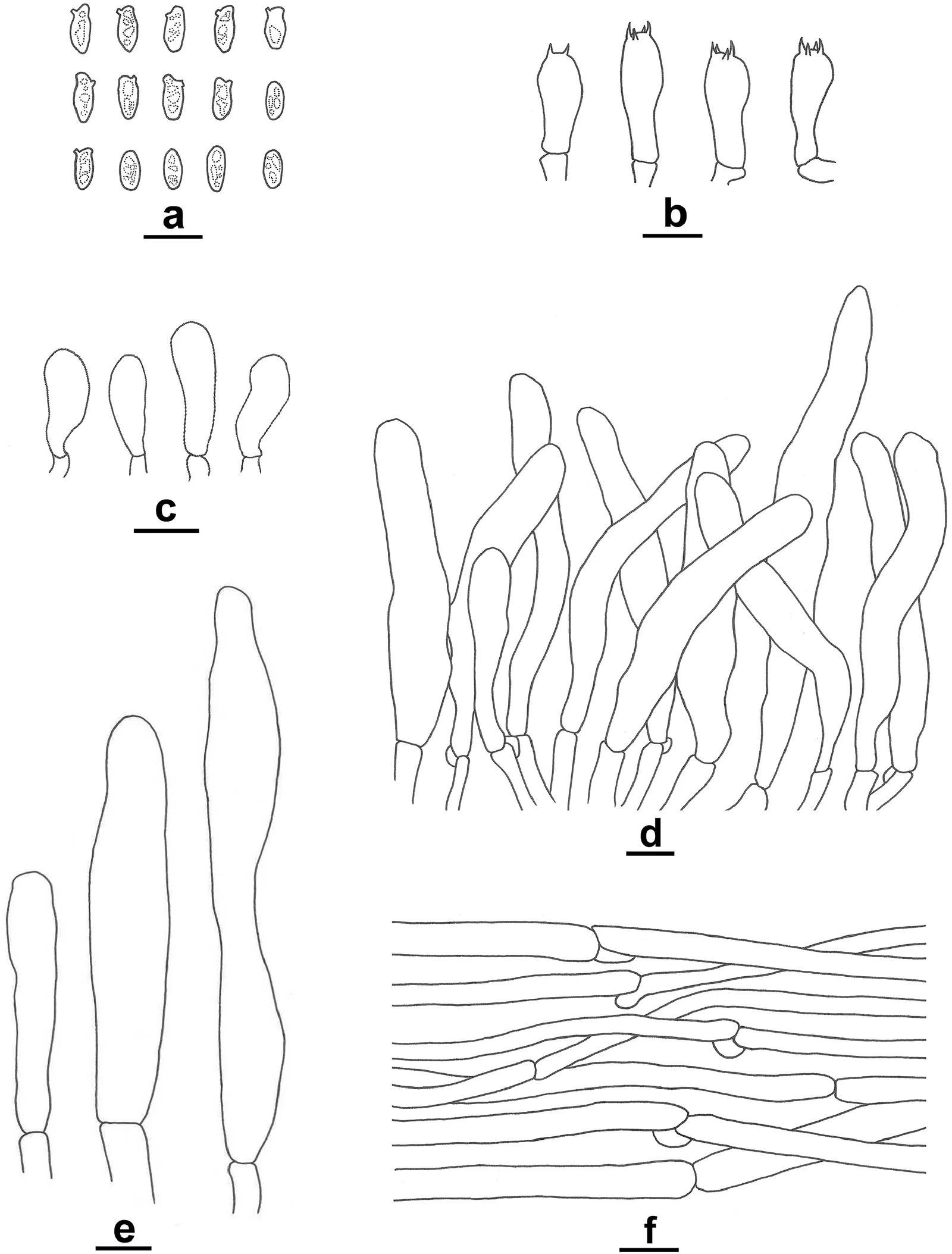
Microscopic structures of *Lepiota maojingbaensis* (holotype, LFNC 0000682). **a**. Basidiospores; **b**. Basidia; **c**. Cheilocystidia; **d**. Terminal cells of pileus covering; **e**. Terminal elements of stipe covering; **f**. Stipe covering hyphae. Line drawings by R. M. Li. Scale bars: 10 μm.

Basidiospores [100/3/2] (5.5–)6.0–8.1(–9.2) × (2.8–)2.9–3.8(–4.1) µm, *X_mm_* = 7.16 ± 0.69 × 3.18 ± 0.27, *Q* = 1.66–2.97, *Qm* = 2.26 ± 0.25, long-ellipsoid to sub-fusiform in front view, sub-triangular in side view, with truncate to spurred base, thin-walled, smooth, hyaline, sometimes with granular contents, dextrinoid, congophilous, cyanophilic, not metachromatic, without suprahilar depression. Basidia 14.0–20.0 × 4.5–7.5 µm, clavate, with guttulate contents, hyaline, thin-walled, 4-spored; sterigmata up to 2.5 µm long. Lamellar trama subregular; parallel to subparallel, rarely branched, hyphae, 3.8–4.5 µm wide, hyaline, thick-walled. Cheilocystidia 13–19 × 6.0–7.5 µm, narrowly clavate to clavate, hyaline, thin-walled. Pleurocystidia absent. Pileus trama interwoven, composed of slightly inflated, septate, 3.1–5.1 µm wide, slightly thick-walled to thick-walled, hyaline hyphae. Pileus covering a trichoderm composed of hyphae 3.0–5.9 μm, thick-walled, terminal elements elongate, narrowly clavate to subcylindrical, 32–65.8 × 8.2–11.5 μm, normally with rounded apex, rarely with tapered apex, occasionally curved, with or without clamps, thin-walled, containing grayish yellow intracellular pigments. Stipe covering a cutis composed of hyphae 3.1–4.9 μm, thick-walled, terminal elements narrowly clavate, with rounded apex or tapering towards apex, 45–192 × 4.5–8 μm, containing pale yellowish intracellular pigments. Clamp connections present in all parts of basidiomata.

##### Habitat and distribution.

Solitary on the ground in mixed coniferous and broad-leaved forests dominated by *Larix principis-rupprechtii*, *Betula platyphylla*, *Quercus* spp., and *Populus davidiana*, Hebei Province, China.

##### Additional specimens examined (paratype).

China • Hebei Province, Chengde City, Longhua County, Maojingba National Forest Park, 41°40'39.15"N, 118°10'39.42"E, 1,450 m asl., 28 July 2019, on ground, leg. Q. An, An 1 (HZC 0000001).

##### Notes.

Phylogenetically, *Lepiota maojingbaensis* is nested within sect. *Stenosporae*, forming a strongly supported lineage (BS = 100%, BPP = 1.00, Fig. 1). It is resolved as a sister group to a subclade comprising *L. boudieri* Bres., *L. aureofulvella* Sysouph., K.D. Hyde, Chukeat. & Vellinga, *L. rhodophylla* Vellinga, and *L. andegavensis* Mornand (Fig. 1). *Lepiota boudieri*, *L. aureofulvella*, and *L. rhodophylla* have a cutis-like pileus covering (Vellinga 2006; Sysouphanthong et al. 2011; Liang 2016), and *L. andegavensis* features longer basidiospores (7.6–10.5 µm; Migliozzi and Donato 2023); these features readily distinguish them from *L. maojingbaensis*.

*Lepiota flavonigrescens* Sarawi & Reschke, *L. hongshiensis* T. Bau & X.Y. Zhou, *L. jilinensis*, *L. sirupa* T. Bau & X.Y. Zhou, and *L. tyrianthina* Sarawi are several recently described species belonging to sect. *Stenosporae* (Suppl. material 2). These species have basidiospores similar in size and shape to those of *L. maojingbaensis*. Nevertheless, they can be distinguished as follows: *L. flavonigrescens* possesses an orange-brown to dark brown pileus with grayish-yellow lamellae (Sarawi et al. 2025b); *L. hongshiensis* has a white pileus with grayish-yellow to olive-brown squamules and poorly differentiated cheilocystidia (Zhou and Bau 2026); *L. jilinensis* features a white pileus adorned with light orange to brownish-orange squamules, as well as larger cheilocystidia (19–35 × 9–18 µm) and terminal cells of the pileus covering (70–306 × 9–19 µm; Zhou and Bau 2026); and *L. sirupa* and *L. tyrianthina* exhibit a distinctly darker-colored pileus surface (Sarawi et al. 2025b; Zhou and Bau 2026).

#### Lepiota varia

Taxon classificationFungiAgaricalesAgaricaceae

M.L. Han, L.A. Wang & Q. An sp. nov.

02CD8198-CF30-5BE0-8F94-6D993FE27809

865587

Figs 2e–h, 5

##### Etymology.

The specific epithet *varia* (from *varius*, Latin) refers to the variable color of pileus and stipe.

##### Holotype.

China • Hebei Province, Zhangjiakou, Zhuolu County, Huangyangshan National Forest Park, 39°55'52"N, 115°07'16"E, 1,300 m asl., 6 September 2023, on ground, leg. L. A. Wang, ZL-1-59 (LFNC 0002558).

##### Diagnosis.

Similar to *L. microstenospora*, but differs in having a cream to flesh-pink pileus with squamules ranging from flesh-pink, brownish vinaceous to rose, smaller cheilocystidia (14–29 × 5.0–9.5 µm), and smaller terminal cells of the pileus covering (32–90 × 6.6–13.3 µm).

##### Description.

Basidiomata small, gregarious. Pileus 29–37 mm in diameter, hemi-spherical to convex, or applanate with distinct umbo; surface dull and dry, cream (4A2/3) to flesh-pink (8A3/9A4) covered with flesh-pink (8A3/9A4), brownish vinaceous (9B4/C5) to rose (13A4/5) squamules at center becoming paler toward the margin; context white (1A1); margin decurved becoming straight at maturity. Lamellae free, moderately close with 1–2 tiers of lamellulae, white (1A1), cream (4A2/3) to straw-yellow (3A/B3); edge fimbriate, concolorous with faces. Stipe 50–65 × 3.5–6 mm, central, cylindrical to subclavate, swelling at base, fibrillose, hollow, with rhizomorphs; surface cream (4A2/3) to clay-buff (6D4), covered by buff (4A4), flesh-pink (8A3/9A4) to fawn (7D/E4) squamules in lower half. Odor not distinctive.

**Figure 5.**
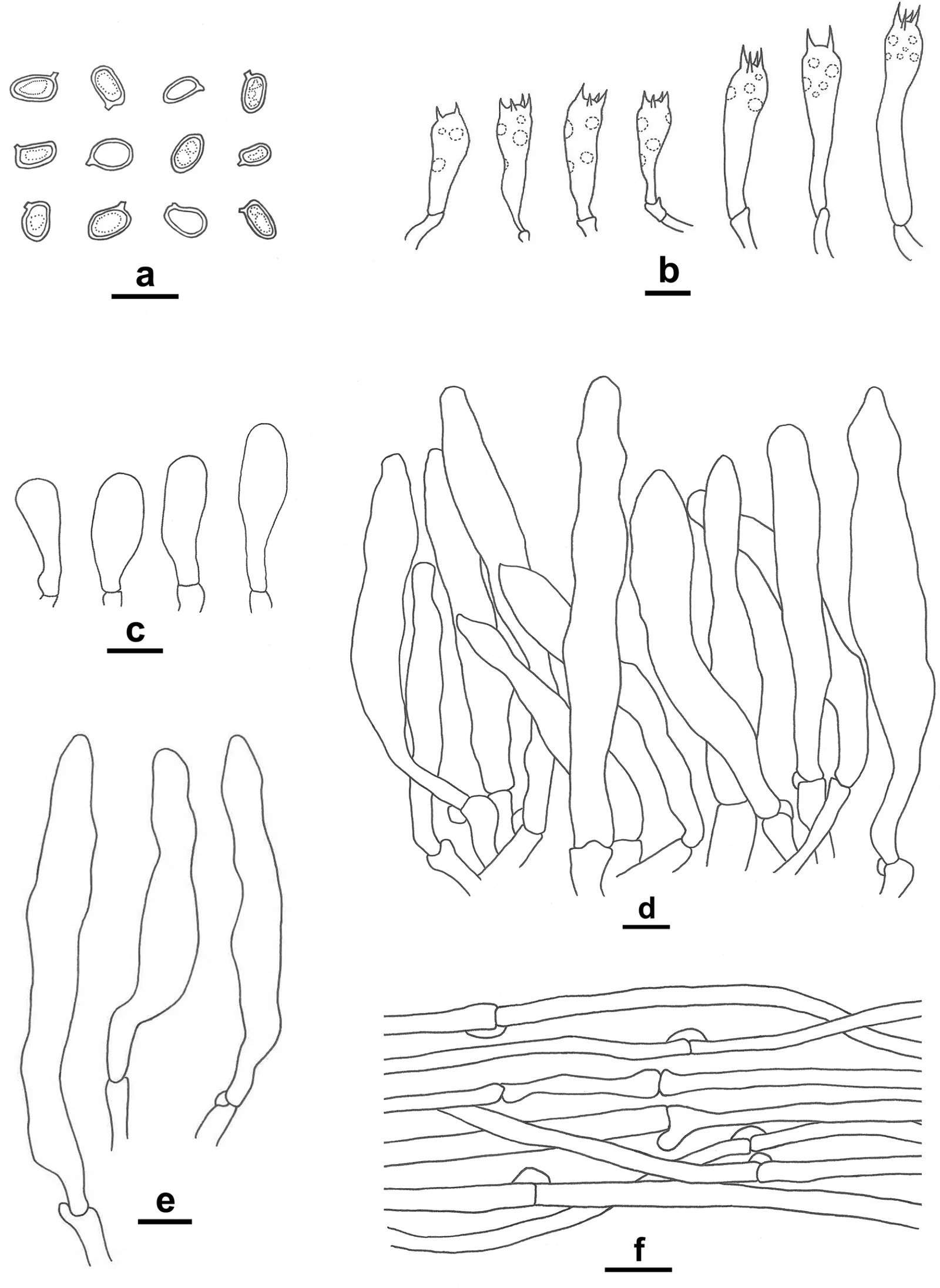
Microscopic structures of *Lepiota varia* (holotype, LFNC 0002558). **a**. Basidiospores; **b**. Basidia; **c**. Cheilocystidia; **d**. Terminal cells of pileus covering; **e**. Terminal elements of stipe covering; **f**. Stipe covering hyphae. Line drawings by R. M. Li. Scale bars: 10 μm.

Basidiospores [100/4/4] (4.0–)5.0–6.1(–7.2) × (2.8–)3.0–4.0(–4.5) μm, *X_mm_* = 5.76 ± 0.67 × 3.53 ± 0.45, *Q* = 1.24–2.33, *Qm* = 1.63 ± 0.20, ellipsoid to long-ellipsoid in frontal view, oblong, sub-triangular or amygdaliform in side view, with truncate to spurred base, thick-walled (0.3–0.5 µm), smooth, hyaline, dextrinoid, congophilous, cyanophilic, not metachromatic, with granular contents, without suprahilar depression and germpore. Basidia 19–41 × 5–10 μm, thin-walled, frequently 4-spored, rarely 2-spored, clavate to broadly clavate, with granular content, hyaline in KOH; sterigmata up to 2.5 µm long. Lamellar trama subregular; hyphae 1.2–3.0 µm wide, hyaline, thick-walled. Cheilocystidia 14–29 × 5.0–9.5 µm, narrowly clavate to clavate, hyaline, thin-walled. Pleurocystidia absent. Pileal trama interwoven, composed of slightly inflated, septate, 3.0–4.3 µm wide, thick-walled to solid, hyaline hyphae. Pileus covering a trichoderm composed of hyphae 1.5–3.2 μm wide, thick-walled, terminal elements erect, narrowly clavate to subcylindrical, 32–90 × 6.6–13.3 μm, normally with rounded apex, rarely with tapered apex, thick-walled to solid, with or without clamp connections, containing pale yellowish intracellular pigments. Stipe covering a cutis made up of hyphae 1.5–3.5 μm wide, parallel, compactly arranged, thick-walled to solid; terminal elements narrowly clavate, with rounded apex or tapering apex, 43–134.1 × 5.8–12.1 μm, containing grayish yellow intracellular pigments. Clamp connections present in all parts of basidiomata.

##### Habitat and distribution.

Solitary on the ground in mixed coniferous and broad-leaved forests dominated by *Quercus* spp., *Betula platyphylla*, *Larix principis-rupprechtii*, and *Pinus tabuliformis*, Hebei province, China.

##### Additional specimens examined (paratypes).

China • Hebei Province, Zhangjiakou, Zhuolu County, Huangyangshan National Forest Park, 39°54'44.11"N, 115°07'22.19"E, 1,300 m asl., 6 September 2023, on ground, leg. L. A. Wang, ZL-2-66 (LFNC 0002559); • Yu County, Xiaowutai National Nature Reserve, 39°53'51"N, 114°59'18"E, 1,350 m asl., 7 September 2023, on ground, leg. X. Zhao, YX-2-18 (LFNC 0002560); • Zunhua, Dangyu Town, Yanjiayu village, 40°11'44.38"N, 117°57'47.56"E, 310 m asl., 5 August 2023, on ground, leg. X. Zhao, QA-33 (LFNC 0002561).

##### Notes.

Phylogenetic analyses place *L. varia* in a well-supported clade in sect. *Stenosporae*, close to *L. citrophylla* (Berk. & Broome) Sacc. and *L. flavonigrescens* Sarawi & Reschke (Fig. 1). Morphologically, *L. citrophylla* and *L. flavonigrescens* are distinguished by larger basidiospores (*L. citrophylla*: 6.0–8.5 × 3.8–4.3 µm, Sysouphanthong et al. 2011; *L. flavonigrescens*: 6.7–7.9 × 3.0–3.6 µm, Sarawi et al. 2025b). *Lepiota aureofulvella* and *L. boudieri* possess basidiospores similar to those of *L. varia*; however, they differ in having a darker-colored pileus surface, a cutis-like pileus covering, and an annulus (Sysouphanthong et al. 2011; Liang 2016).

Several recently described species in sect. *Stenosporae* (Suppl. material 2), *L. hongshiensis*, *L. microstenospora*, *L. brunneoolivacea*, and *L. flavostipitata*, share similar basidiospore dimensions and shapes with *L. varia*; however, they differ in pileus coloration and micromorphological features. *Lepiota hongshiensis* is distinguished by its white pileus with grayish-yellow to olive-brown squamules and undifferentiated cheilocystidia; *L. microstenospora* is characterized by light orange to brownish-orange squamules and larger cheilocystidia (23–38 × 5–9 µm) and terminal cells of the pileus covering (89–247 × 6–15 µm) (Zhou and Bau 2026). *Lepiota brunneoolivacea* has a light yellowish-brown to dark yellow pileus, whereas *L. flavostipitata* features a white pileus covered with light orange to reddish-brown or brown squamules (Sarawi et al. 2025b).

#### Lepiota xiaowutaishanensis

Taxon classificationFungiAgaricalesAgaricaceae

M.L. Han, L.A. Wang & Q. An sp. nov.

D9C86941-459A-530F-AA62-0035DF6F947D

865588

Figs 2i, 6

##### Etymology.

The specific epithet *xiaowutaishanensis* (Latin) refers to the location of the species in Xiaowutaishan National Nature Reserve.

##### Holotype.

China • Hebei Province, Zhangjiakou, Yu County, Xiaowutaishan National Nature Reserve, 39°46'20.36"N, 114°51'48.33"E, 1,350 m asl., 7 September 2023, on ground, leg. L. A. Wang, YX-2-7 (LFNC 0002562).

##### Diagnosis.

Similar to *Lepiota psalion* Huijser & Vellinga, but differs in a white, flesh-pink to clay-pink pileus and a larger stipe (60–88 × 3–6 mm).

##### Description.

Basidiomata small, gregarious. Pileus 1.8–2.5 cm in diameter, conical to parabolic when young, becoming convex at maturity; surface dull and dry, white (1A1), flesh-pink (8A3/9A4) to clay-pink (6B/C4), smooth; context white (1A1), soft, thin ca. 3-4 mm; margin decurved becoming straight at maturity. Lamellae free, crowded with 1–3 tiers of lamellulae, white (1A1) to clay-pink (6B/C4); margin entire and slightly wavy. Stipe 60–88 × 3–6 mm, central, cylindrical, equal at base, fibrillose, hollow; white (1A1), flesh-pink (8A3/9A4) to fawn (7D/E4). Volva absent. Odor not distinctive.

**Figure 6.**
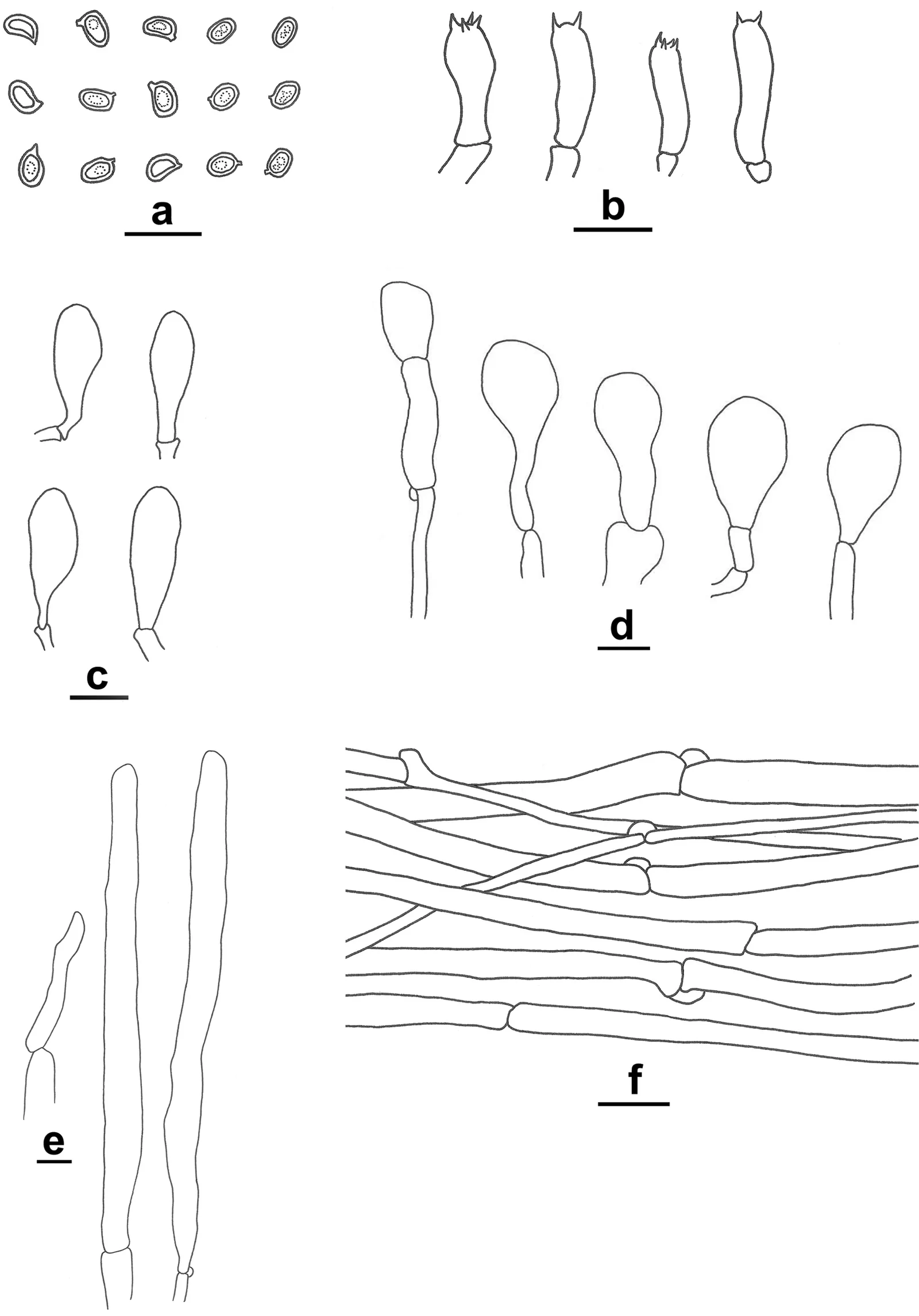
Microscopic structures of *Lepiota xiaowutaishanensis* (holotype, LFNC 0002562). **a**. Basidiospores; **b**. Basidia; **c**. Cheilocystidia; **d**. Terminal elements of pileus covering; **e**. Terminal elements of stipe covering; **f**. Stipe covering hyphae. Line drawings by R.M. Li. Scale bars: 10 μm.

Basidiospores [100/3/2] (3.0–)3.5–5.0(–5.1) × (2.0–)2.3–3.5(–4.0) µm, *Xmm* = 4.37 ± 0.47 × 2.94 ± 0.33, *Q* = 1.17–1.96, *Qm* = 1.50 ± 0.17, thick-walled (0.3–0.6 µm), ellipsoid in frontal view, ellipsoid, broadly ellipsoid to oblong in side view, some slightly truncate, hyaline, smooth, with refractive guttules, dextrinoid, congophilous, cyanophilic, not metachromatic, without suprahilar depression and germpore. Basidia 13–21 × 4–6.5 μm, thin-walled, frequently 4-spored, rarely 2-spored, clamped, clavate, hyaline in KOH. Lamellar trama subregular; hyphae 2.1–4.1 µm wide, hyaline, thick-walled. Cheilocystidia 20.1–28.9 × 5.0–10.0 µm, clavate to broadly clavate, smooth, thin-walled, hyaline in KOH. Pileal trama interwoven, composed of slightly inflated, septate, 2.5–4.0 µm wide, thick-walled to solid, hyaline hyphae. Pileus covering a hymeniderm composed of erect, broadly clavate or narrowly clavate elements 13.3–43.5 × 9.8–20.8 um, thick-walled, with or without clamp connections, containing grayish yellow intracellular pigments. Stipe covering a cutis composed of hyphae 3.1–5.1 μm wide, parallel, compactly arranged, thick-walled to solid, occasionally branched, hyaline or yellowish, with or without clamp connections; terminal elements extremely narrowly clavate, with rounded apex, 61.9–183.2 × 5.4–13.2 μm, containing green-yellow intracellular pigments. Clamp connections present in all parts of basidiomata.

##### Habitat and distribution.

Solitary on the ground in mixed coniferous and broad-leaved forests dominated by *Pinus tabuliformis* and *Betula platyphylla*, Hebei Province, China.

##### Additional specimens examined (paratype).

China • Hebei Province, Zhangjiakou, Yu County, Xiaowutaishan National Nature Reserve, 39°46'25.12"N, 114°51'54.85"E, 1,400 m asl., 7 September 2023, on ground, leg. M. L. Han, Han 5 (LFNC 0002563).

##### Notes.

Phylogenetic analysis shows that *L. xiaowutaishanensis* is closely related to *L. thiersii* Sundb., *L. recondita* Tatti, Huijser & Vizzini, and *L. sinorecondita*, forming a distinct cluster (Fig. 1). Morphologically, all four species share similarly sized basidiospores. However, in contrast to *L. xiaowutaishanensis*, the other three species exhibit dark pilei and non-dextrinoid basidiospores (Sundberg 1989; Vizzini et al. 2019). *Lepiota psalion* has basidiospores similar in size and shape, a hymeniform pileus covering, and clavate cheilocystidia similar to those of *L. xiaowutaishanensis*. However, it can be distinguished by its cream to pinkish-light brown pileus with concentric, non-uplifted squamules and a smaller stipe (22–33 × 1.5–2 mm; Vizzini et al. 2019).

#### Lepiota sect. Paralilaceae

Taxon classificationFungiAgaricalesAgaricaceae

(Bon) M.L. Han, L.A. Wang & Q. An comb. nov.

1C54F17B-C1FD-5B95-A994-CBD94F402175

865673

##### Basionym.

*Lepiota* subsect. *Paralilaceae* Bon, Doc. Mycol. 22(no. 88): 30. 1993.

##### Type species.

*Lepiota ochraceofulva* P.D. Orton.

##### Notes.

The species *L. ochraceofulva*, *L. bengalensis* Hosen & T.H. Li, *L. chiangraiensis* Sysouph., Thongkl. & K.D. Hyde, and *L. beninensis* Sarawi & Manz, previously placed in subsect. *Paralilaceae* (Sarawi et al. 2025a), were found to form a separate section in the current study. The name sect. *Paralilaceae* has therefore been adopted for this lineage, maintaining consistency with the original taxonomic concept. The species of sect. *Paralilaceae* generally have basidiomata with vivid orange-brown pilei and dextrinoid, ellipsoid basidiospores that are more than 5 um long (Sarawi et al. 2025a).

## Discussion

The genus *Lepiota* is one of the most species-rich and taxonomically challenging groups in *Verrucosporaceae* due to morphological similarities, historical classification instability, and the polyphyly of some traditional sections (Vellinga 2003; Hou and Ge 2020; Sarawi et al. 2025a). Recent phylogenetic studies have clarified the infrageneric relationships and validated several sections within *Lepiota* (Sarawi et al. 2025a, 2025b). Based on integrated evidence from multigene phylogeny and morphology, this study describes four new *Lepiota* species assigned to three sections: sect. *Eriophorae*, sect. *Stenosporae*, and sect. *Cristatae*. These taxa are firmly placed within their respective sections, consistent with the updated classification framework proposed by Sarawi et al. (2025a). All four new species align well with the common morphological features established for these sections. Specifically, *L. huangyangshanensis* (sect. *Eriophorae*) is characterized by a pileus covering composed of chains of spherical or cylindrical cells and small ellipsoid to amygdaliform basidiospores; *L. maojingbaensis* and *L. varia* (sect. *Stenosporae*) exhibit a trichodermal pileus covering and truncate to spurred basidiospores; and *L. xiaowutaishanensis* (sect. *Cristatae*) features a hymeniform pileus covering and ellipsoid, broadly ellipsoid to oblong basidiospores. These congruent morphological patterns thus consistently support their phylogenetic placement within the sectional frameworks outlined by Sarawi et al. (2025a).

### Phylogenetic comparisons with other studies

The phylogenetic relationships among the eight sections are mostly consistent between the current study and Sarawi et al. (2025a), with the exception of the clade comprising *Lepiota lilaceostriata* Sarawi and *L. lilacea* Bres. In Sarawi et al. (2025a), *Lepiota* sect. *Lilaceae* was formed by two subsections: subsect. *Lilaceae* (with its type species *L. lilacea* and *L. lilaceostriata*) and subsect. *Paralilaceae*. The latter subsection differs from subsect. *Lilaceae* in the dextrinoidity of the spores, basidiomatal colors, and basidiospore sizes, while sharing a hymeniform pileus covering (Sarawi et al. 2025a). In the current study, *L. ochraceofulva* and three further species formed a distinct, highly supported lineage (BS = 100%, BPP = 1.00), corresponding to sect. *Paralilaceae*. Meanwhile, *L. lilaceostriata* and *L. lilacea* were recovered as a well-supported clade (BS = 100%, BPP = 1.00) that is sister to sect. *Lepiota*. Morphologically, although *L. lilaceostriata* and *L. lilacea* exhibit a dark-colored pileus, a dark underside of the annulus, and small, ellipsoid basidiospores consistent with sect. *Lepiota*, their non-dextrinoid basidiospores, and hymeniform pileus covering represent a notable divergence from the typical characteristics of this section (Sarawi et al. 2025a). Consequently, the sectional assignment of these two species warrants further investigation.

Compared with the three-gene phylogeny (ITS + nrLSU + *RPB2*) reported by Sarawi et al. (2025b), the four-gene dataset (ITS + nrLSU + *RPB2* + mtSSU) in the current study yielded a congruent topology regarding the terminal clades (sect. *Stenosporae*, sect. *Helveolae*, and sect. *Cristatae*) but revealed significant rearrangements in the basal and intermediate backbone relationships of *Lepiota*. In Sarawi et al. (2025b), sect. *Eriophorae* was recovered as sister to the upper terminal clades. Their results showed that sections *Lepiota*, *Fuscovinaceae*, and *Lilaceae* formed a major clade, within which sections *Lepiota* and *Fuscovinaceae* were sister to each other, with sect. *Lilaceae* as a neighboring lineage. In contrast, the current study identified sect. *Fuscovinaceae* as sister to the terminal clades. Despite some topological variability, both ML and BI analyses supported sect. *Lilaceae* as sister to sect. *Lepiota*. Furthermore, subsect. *Paralilaceae* did not form a monophyletic clade within sect. *Lilaceae*; consequently, it was raised to the rank of section. Additionally, sect. *Eriophorae* was placed as a basal clade within *Lepiota*.

### Comparisons with other *Lepiota* species from China

*Lepiota maojingbaensis* and *L. varia* were compared with the 15 previously recorded taxa of *Lepiota* sect. *Stenosporae* from China (Yang et al. 2019; Zhou and Bau 2026). They differ primarily in pileus characteristics, cheilocystidia, and basidiospores. Among these, *L. luteocastanea* E. Horak (Zhou and Bau 2026) and *L. subcitrophylla* Hongo are the only two species that currently lack molecular data. However, they can be distinguished from *L. maojingbaensis* and *L. varia* by several key features: *L. luteocastanea* is characterized by its red-brown to wine-red squamulose pileus and golden-yellow lamellae (Horak 1980), whereas *L. subcitrophylla* differs in having a pale yellow context and significantly larger basidiospores (8.5–10.5 × 3.5–4 µm; Hongo 1956).

There are three species within sect. *Eriophorae* in China: *L. jacobi* Vellinga & Knudsen, *L. leprica* (Berk. & Broome) Sacc., and *L. ompnera* (Berk. & Broome) Zhu L. Yang (Yang et al. 2019). Among these, *L. jacobi* can be easily distinguished from *L. huangyangshanensis* in the phylogenetic tree (Fig. 1). After consulting the literature and the NCBI database, no molecular data were found for *L. leprica* and *L. ompnera*. However, morphologically, they can be distinguished from *L. huangyangshanensis* (Yang et al. 2019). *Lepiota leprica* possesses larger basidiospores (6.0–8.0 × 3.5–4.5 µm) and a pileus surface composed of chains of cylindrical cells. *Lepiota ompnera* has brown to gray-brown squamules covering a dirty brown to yellowish-brown pileus surface and a brownish to dull white stipe.

Yang et al. (2019) recorded seven taxa within *Lepiota* sect. *Lilaceae* in China, which has recently been subdivided into sect. *Lilaceae* and sect. *Cristatae* (Sarawi et al. 2025a). Among them, molecular sequences clearly distinguish *L. xiaowutaishanensis* from five taxa. However, two species have no available molecular sequences: *L. apalochroa* (Berk. & Broome) Sacc. and *L. micropholis* (Berk. & Broome) Sacc. Morphologically, compared with *L. xiaowutaishanensis*, *L. apalochroa* has white to pale beige basidiomata with brown to brown-yellow squamules and spherical to broadly elliptical terminal elements of the pileus covering containing brown to pale brown intracellular pigments; and *L. micropholis* has white to off-white basidiomata with gray to gray-brown squamules and pear-shaped to nearly globose terminal elements of the pileus covering containing gray-brown intracellular pigments (Yang et al. 2019).

### Comparisons with other *Lepiota* species from outside of China

As *Lepiota* species are distributed nearly worldwide, studies from other regions were also thoroughly reviewed (Massee 1899; Locquin 1951; Pegler 1972; Horak 1980; Bon 1981; Candusso and Lanzoni 1990; Aberdeen 1992; Akers and Sundberg 2001; Cho and Ban 2008; Sysouphanthong et al. 2011), with an emphasis on taxa lacking DNA sequence data. Despite the difficulty of achieving exhaustive coverage of the entire genus, morphologically similar species are compared below to emphasize the distinctive characters of the new taxa.

While *Lepiota leontoderes* (Berk. & Broome) Sacc., *L. pyrrhaes* (Berk. & Broome) Sacc., and *L. mesomorpha* (Bull.) Gillet in sect. *Stenosporae* are currently classified under *Agaricus* L. in MycoBank; they remain accepted members of *Lepiota* according to Index Fungorum. These three species share basidiospore dimensions with *L. varia* but differ in pileus features: *L. leontoderes* (type locality: Ceylon) has a dull tawny-brown to fuscous pileus with whitish squamules (Pegler 1972); *L. pyrrhaes* (type locality: Ceylon) has smaller basidiomata with a brick-red to orange pileus (Pegler 1972); and *L. mesomorpha* (type locality: France) has a glabrous, yellowish to pale yellow-brown pileus (Massee 1899). Additionally, *L. aurora* Murrill, *L. pyrrhaes*, and *L. pseudofelina* J.E. Lange overlap in spore size with *L. maojingbaensis*. However, the Australasian *L. aurora* exhibits a conspicuously orange or red-orange pileus with concolorous squamules and fibrils and distinctly larger cheilocystidia (25–45 × 4–7 µm; Horak 1980); *L. pyrrhaes* bears a brick-red to orange pileus surface with verrucose squamules and a reddish-brown stipe (Pegler 1972); and *L. pseudofelina* has a white, grayish to dark fuliginous pileus with brown squamules (Bon 1981).

*Lepiota microspila* (Berk.) Sacc. (sect. *Eriophorae*), reported from Ceylon, shares similarly sized basidiospores with *L. huangyangshanensis* but differs in having an ochraceous tawny to rufous pileus surface, a pale ochraceous tawny stipe covered with brown strigose fibrils, and markedly smaller basidia (6.5–8 × 3–3.5 µm; Pegler 1972). Another member of sect. *Eriophorae*, the American *L. subflavescens*, is distinguished from *L. huangyangshanensis* by its larger basidiospores (6–7 × 3–4 µm; Murrill 1914). *Lepiota oenocephala* (Berk. & Br.) Sacc. (sect. *Cristatae*), which is currently synonymized under *Agaricus* L. in MycoBank but retained in *Lepiota* in Index Fungorum, was described from Ceylon and shares similarly sized basidiospores with *L. xiaowutaishanensis*. However, it differs in having an orange-vinaceous to ‘Etruscan Red’ pileus with cracked squamules, a bright yellow context in the pileus, and smaller basidia (8–10 × 2.5–3 µm; Pegler 1972).

### Discussion and future perspectives on *Lepiota* in northern China

Mao et al. (2023) described *Lepiota atrobrunneodisca* L. Fan, L. Xia & N. Mao, a new species in sect. *Cristatae*, from Shanxi Province in northern China. Zhou and Bau (2026) conducted a survey of *Lepiota* in the temperate forests of Northeast China, documenting six new species and one new record belonging to sect. *Stenosporae*. However, systematic sampling across the entire temperate zone of northern China remains fragmentary, and large-scale biogeographic comparisons are currently constrained by limited specimen-based occurrence records and by the absence of sequence data for all species recorded from the area.

Although the precise ecological roles of *Lepiota* species have not been fully elucidated, they are widely recognized as saprotrophic fungi with potential functions in forest litter decomposition and nutrient dynamics. The four new taxa described herein further enrich our knowledge of *Lepiota* species diversity in northern China and provide baseline morphological and molecular data for future taxonomic, ecological, and biogeographic investigations of this genus.

## Supplementary Material

XML Treatment for Lepiota huangyangshanensis

XML Treatment for Lepiota maojingbaensis

XML Treatment for Lepiota varia

XML Treatment for Lepiota xiaowutaishanensis

XML Treatment for Lepiota sect. Paralilaceae

## Additional information

### Artificial Intelligence (AI) use

The authors accept full responsibility for the content of the manuscript, including the disclosure of any use of AI.

No AI tools were used in the preparation of this manuscript.

### Author ORCIDs

Q. An https://orcid.org/0000-0001-5917-2497

X.-Y. Zhao https://orcid.org/0009-0001-2210-632X

R.-M. Li https://orcid.org/0009-0009-1140-8187

W.-R. Shui https://orcid.org/0009-0004-1826-6883

W.-B. Zhang https://orcid.org/0009-0001-7080-0500

Y.-M. Liu https://orcid.org/0009-0005-7627-1338

Y.-J. Zhang https://orcid.org/0009-0002-0306-6155

L.-A. Wang https://orcid.org/0000-0003-0318-9956

X. Zhao https://orcid.org/0009-0003-4851-8538

M.-L. Han https://orcid.org/0000-0003-4936-3083

### Data availability

All of the data that support the findings of this study are available in the main text or Supplementary Information.

## Supplementary materials

Supplementary material 1 Bayesian inference tree Data type jpg Explanation note Bayesian inference tree (ITS + nrLSU + RPB2 + mtSSU). Branches are labeled with Bayesian posterior probabilities > 0.95. New species and new sequences are in bold. https://binary.pensoft.net/file/1821678This dataset is made available under the Open Database License (http://opendatacommons.org/licenses/odbl/1.0/). The Open Database License (ODbL) is a license agreement intended to allow users to freely share, modify, and use this Dataset while maintaining this same freedom for others, provided that the original source and author(s) are credited. Qi An, Xi-Ya Zhao, Rui-Min Li, Wan-Rui Shui, Wen-Bo Zhang, Yi-Man Liu, Yu-Jiao Zhang, Li-An Wang, Xuan Zhao, Mei-Ling Han

Supplementary material 2 Maximum likelihood (ML) tree Data type jpg Explanation note Maximum likelihood (ML) tree illustrating the phylogeny of *Lepiota* species and the outgroup based on ITS sequences, incorporating new species described by Zhou and Bau (2026) and Li et al. (2026). Branches are labeled with maximum likelihood bootstrap support ≥ 50% and Bayesian posterior probabilities > 0.95. https://binary.pensoft.net/file/1821679This dataset is made available under the Open Database License (http://opendatacommons.org/licenses/odbl/1.0/). The Open Database License (ODbL) is a license agreement intended to allow users to freely share, modify, and use this Dataset while maintaining this same freedom for others, provided that the original source and author(s) are credited. Qi An, Xi-Ya Zhao, Rui-Min Li, Wan-Rui Shui, Wen-Bo Zhang, Yi-Man Liu, Yu-Jiao Zhang, Li-An Wang, Xuan Zhao, Mei-Ling Han
